# Skills2Care: An innovative, cooperative learning programme for community health workers in South Africa

**DOI:** 10.4102/phcfm.v13i1.2922

**Published:** 2021-10-21

**Authors:** Therese M. Boulle, Paul Cromhout, Khuzwayo August, Dave Woods

**Affiliations:** 1 Small Projects Foundation, East London, South Africa; 2 Bettercare, Cape Town, South Africa

**Keywords:** community health worker, community health worker training, community health worker programmes, lay health worker, maternal and child health, access to health care, village health worker, community care worker

## Abstract

**Background:**

Community health workers (CHWs) hold potential to support universal health coverage and better health for vulnerable communities. They are integral to the re-engineered Primary Health Care (PHC) strategy, introduced in South Africa in 2011. This study focussed on how to train CHWs in large numbers, especially in resource-limited, rural settings. Skills2Care, a method of cooperative learning for CHWS, has been pioneered in the Eastern Cape of South Africa.

**Aim:**

To determine whether Skills2Care could improve the cognitive knowledge of CHWs; to understand their response and attitude to the programme; to explore factors that enabled and inhibited learning and to consider its viability as a training method.

**Setting:**

Research was conducted in 2019 in the Ngqeleni subdistrict of the O.R. Tambo district, in rural Eastern Cape.

**Methods:**

A group-learning model using specifically tailored study modules in booklet format, addressing mother and baby care, was used. A facilitator promoted learning. Knowledge assessment was conducted by pre- and post-study testing using multiple choice questions. Focus group discussions and interviews explored the appropriateness and acceptability of this method, and factors enabling and inhibiting the learning.

**Results:**

This method of peer group cooperative learning can significantly increase the cognitive knowledge of CHWs. Test scores indicated a significant (13%) improvement. Focus group discussions indicated that participants valued this method as it increased knowledge and boosted their confidence.

**Conclusion:**

This innovative approach to district-based, continuing education suggests that CHWs could be trained in large numbers without the need for additional resources.

## Introduction

Community health workers (CHWs), holding the potential to support universal health coverage, offer hope of better health for vulnerable communities.^1,2,3^ They provide an opportunity for equitable access to safe, primary health services.^4^ Community health workers have been described as members of the community who ‘provide basic health care within their community, and are capable of providing preventive, promotional and rehabilitation care to that community’.^5^

Several recent systematic reviews have indicated that training and continuous training for CHWs are important if their work is to remain relevant and up-to-date.^1,2,3,6^ Murphy et al.^6^ have pointed to the lack of opportunities for professional development for CHWs, which training seeks to address.^7^ The systematic reviews also indicated that training is necessary to improve CHWs’ motivation, and increase community confidence in their services.^1,2^

Refining the focus on the question of how training is implemented, especially in remote, under-served rural areas, has seemingly not been documented. This research deals with testing and identifying a method of providing knowledge and skills to CHWs to improve healthcare services to communities in resource-poor contexts. Few methods have been proposed to date which are sustainable and can be rolled out rapidly, without large costs and which can provide increases in knowledge and skills of CHWs. Traditionally, continuing education for health workers, including CHWs consists of courses run by formal trainers at large central hospitals. These courses are often expensive to run and far from health workers’ families and places of work, making them inaccessible.^8^

This study was conducted to determine the appropriateness of the Skills2Care learning model in improving knowledge and understanding for CHWs, so as to improve community care in rural areas. The Skills2Care content that was tested related to perinatal care of the mother and baby, and included four modules. These comprised: care of the mother, the newborn baby, exclusive breastfeeding and human immunodeficiency virus (HIV) in mother and baby. The programme is a collaboration amongst Small Projects Foundation, the Perinatal Education Programme, Bettercare and Kistefos funding agency.

Skills2Care is a learning programme modelled on the innovative Perinatal Education Programme for doctors, professional nurses and midwives, which has been shown to be highly successful.^9^ Results from pre- and post-tests, with 114 midwives, demonstrated significant improvement in cognitive knowledge and understanding. In this earlier study, group-based cooperative learning without the support of a formal trainer was achieved, sparking hope that an adaptation of the programme for CHWs may be equally fruitful.

Skills2Care, being decentralised and supported through a modularised approach on-site, seemed to offer an alternate method for CHW training. Hence, this study intended to determine the effectiveness of the decentralised and facilitated learning method, to determine CHWs’ response to it and to understand the factors affecting their learning. The term ‘learning’ was chosen as CHWs’ active engagement with the learning material was required. It supported the idea of an active learning process, different from their training or teaching which may imply a more passive recipience of training.

Policy for Ward Based Primary Healthcare Outreach Teams in South Africa indicates that the country needs approximately 55 000 CHWs.^10^ According to Pillay and Barron,^11^ health outcomes related to CHWs in South Africa (SA) are generally accepted to be sub-optimal, especially in the areas of maternal and child health. The reasons advanced include: inadequate training, inadequate support and supervision. Posing the question of how large numbers of CHWs can be trained, especially in resource-limited settings, is imperative to ensure that they have the requisite knowledge and confidence to be effective. Given the number of CHWs that a primary health care (PHC) system ought to have, it is essential to give consideration to effective and affordable strategies for on-site training.

## Methods

### Study design

The study included both qualitative and quantitative components.

The quantitative component comprised a pre-and post-study design to assess knowledge. For each of the four modules, a multiple-choice pre-test was conducted before the learning process started, and a post-test, with the same set of multiple-choice questions, was conducted at the end of the learning process. The results of the pre-test documented CHW knowledge related to the topic prior to learning, whilst the post-test assessed their knowledge after learning. The difference between the two results provided an indication of knowledge gained.

The qualitative research component used a multi-method approach, consisting of focus group discussions and interviews with key stakeholders. Where required, focus group discussions were translated into CHW home language, isiXhosa, with the support of the facilitator.

Semi-structured interviews were held with four CHWs, the director of Small Projects Foundation (SPF), the programme manager who manages and oversees the programme and the facilitator who implemented the programme. These included face-to-face and telephonic interviews. Questions for the semi-structured interviews and focus group discussions probed attitudes to the learning programme, the perceived value of the learning process and the factors which enabled and inhibited the acquisition of knowledge for the participants.

### Setting

Ten clinics in the Ngqeleni sub-district of the O.R. Tambo District, Eastern Cape, South Africa, participated in the study. These clinics, all in rural areas, included: Buntingville, Canzibe, Lujizweni, Maqanyeni, Ngqeleni, Nkumandeni, Nqanda, Ntaphane, Ntibane and Pilani.

### Study population

The study population comprised 63 CHWs: 49 from SPF, 4 from Siyakhanyisa (an NGO) and 10 from the Department of Health (DoH). Participants were required to complete a registration form with demographic details.

Table 1 provides the demographic characteristics of the participants: depicting CHW ages, gender, length of service, educational levels and some household information.

**TABLE 1:** Demographic characteristics of Community health workers (*n* = 63).

Variable	Participants	
	*n*	%
**Age**		
< 26 years	10	15.9
26–35 years	25	39.7
36–45 years	21	33.3
> 45 years	7	11.1
**Gender**		
Men	8	12.7
Women	55	87.3
**Length of service**		
< 1.5 years	23	36.5
1.5 year	24	38.1
2 years	1	1.6
3 years	1	1.6
4 years	6	9.5
5 years	4	6.3
Unknown	4	6.3
**Educational level**		
ABET Level 4	2	3.2
< Grade 12	16	25.4
Grade 12	43	68.3
Diploma	2	3.2
**Number of people living in CHW households**		
< 5 people	15	23.8
5–8 people	29	46.0
9–12 people	14	22.2
> 12 people	5	7.9
**Breadwinners**		
CHW is the breadwinner	49	77.8
CHW is not the breadwinner	14	22.2
**Households in receipt of social grants**		
Households with social grants (average 2.5 grants per household)	55	87.3
Households without social grants	8	12.7
**Households and additional salaries**		
Households with additional salaries	18	28.6
Households without additional salaries	45	71.4

ABET, adult basic education and training; CHW, community health worker.

### Intervention

The study manual, *Mother and Baby Care for Community Health Workers* written by Prof David Woods from Bettercare, provided the content for the learning. The manual is one of a series of learning programmes for healthcare workers supporting distance-learning (https://bettercare.co.za/learn). It is intended to provide ‘appropriate, affordable and up-to-date learning material for health care workers so that they can continue to learn, practice and deliver excellent patient care’.^8^ Four modules included in the manual are used in this study: Care of the Mother, Care of the Newborn baby, Exclusive Breastfeeding and HIV in Mother and Baby.

Each module follows a standard format with learning objectives clearly stated at the outset, followed by its content using a question-and-answer, problem-solving style of learning, and thereafter a set of case studies, providing practical examples and applying module knowledge to real-life situations. Each module has a self-assessment test comprising 10 multiple-choice questions to determine knowledge after the module is studied.

All participants were provided with a copy of the study manual. The details of the study, its purpose and the standardised process for learning were explained to the participants by the facilitator. The facilitator was an adult educator, employed by SPF who has extensive experience working with CHWs. From previous experience, it had been learnt that the deployment of a facilitator to support CHW learning produced better results than a purely self-study method. The main role of the facilitator was to encourage and support group learning rather than to teach. The CHWs shared and discussed newly learnt knowledge and understanding in a spirit of cooperative learning and peer tuition.

Learning groups were established by CHWs at the clinics where they were based. Active participation was encouraged. Ten learning groups were established, one at each facility. The facilitator travelled to each group promoting learning.

Each week presented a different component of the learning process:

- In week one, the pre-test was written and the material from the first module was read together by the group and discussions of the content were advanced by the facilitator. The CHEWs were encouraged to read and learn the material at home.
- In week two, CHWs returned to the group, where any challenges with the learning material were discussed and clarified. Case studies from the manual, in question-and-answer style format, were presented. Community health workers were encouraged to share their own relevant case experiences. They returned home to complete their self-study of the material.
- In week three, the post-test (the same as the pre-test) was conducted. The next module started with the learning cycle repeating itself for each module.

Where possible, CHWs with smart phones established WhatsApp groups through which they communicated with one another, asking questions about the study material and supporting one another.

The completed pre-test and post-tests were sent to the researcher to be marked and recorded. Results for the pre- and post-tests were recorded on Excel spreadsheets and analysed. In order to pass, a minimum result of 80% was required for the post-test.

### Data collection

#### Quantitative data

Quantitative data were collected for the pre- and post-tests. A total of 63 participants completed the pre-tests and the post-tests for the following modules: Care of the Mother and Care of the Newborn Baby. The Exclusive Breastfeeding post-test was completed by 60 CHWs and 58 completed the HIV in Mothers and Babies post-test. The number of CHWs reduced with CHWs leaving the programme to pursue other opportunities.

#### Qualitative component

The researcher spent a week in the Ngqeleni sub-district, prior to the study, where introductions were made to the CHWs and the study. She also spent a week after the study and held interviews with four CHWs, the facilitator, programme manager as well as two professional nurses from participating clinics working for the DoH. Five focus group discussions were held with 48 CHWs: two were held two weeks after the start of the process and three were held on its completion.

The interviews and focus group discussions were held at the clinics. They were recorded, transcribed and analysed.

### Analysis

#### Quantitative

Scores for the pre- and post-tests for each participant in each module were captured on an Excel spreadsheet. The average scores for each module were calculated along with the total average for all the modules. Participants’ scores of pre-and post-tests were compared.

#### Qualitative

An analysis of the data was collated from the interviews and focus group discussion. Key recurring themes were elicited by looking for patterns of shared meaning across the data and coding these accordingly.

### Ethical considerations

Ethics approval was provided by Human Sciences Research Council: Protocol number REC7/22/08/18 on 28 September 2018. Small Projects Foundation signed a memorandum of understanding with the Provincial Department of Health, which provides for implementation and study of the Skills2Care programme.

All participants were provided with an information sheet, which described the study and gave contact details of the researcher and SPF managers. The study process, its ethical requirements as well as their voluntary participation and confidentiality were explained. Any clarifications or questions were answered. The CHWs then completed the consent form, providing their agreement to voluntarily participate in the study.

In an effort to maintain anonymity, all participants were provided a unique number that they used throughout the process for both pre- and post-tests, thus no names were utilised. The researcher used the unique numbers to match the results to the participants.

## Results

### Results quantitative

The results of pre- and post-tests for each module are presented in Table 2, with the descriptive statistics displayed.

**TABLE 2:** Results per module for the pre- and post-tests.

Module (pre- and post-test)	*n*	Mean	s.d.	Minimum	Quartile 1	Median	Quartile 3	Maximum
Pre: Care of the mother	63	80.32	13.19	30.00	70.00	80.00	90.00	100.00
Post: Care of the mother	63	83.33	12.95	30.00	80.00	80.00	90.00	100.00
Difference: Care of the mother	63	3.02	17.66	−60.00	−10.00	0.00	10.00	50.00
Pre: Care of the newborn	63	54.13	10.72	30.00	50.00	50.00	60.00	80.00
Post: Care of the newborn	63	87.46	10.62	50.00	80.00	90.00	100.00	100.00
Difference: Care of the newborn	63	33.33	13.91	−10.00	30.00	30.00	40.00	60.00
Pre: Exclusive breastfeeding	63	86.35	15.17	40.00	80.00	90.00	100.00	100.00
Post: Exclusive breastfeeding	60	92.67	8.41	60.00	90.00	90.00	100.00	100.00
Difference: Exclusive breastfeeding	60	6.17	12.50	−10.00	0.00	5.00	10.00	40.00
Pre: HIV in mother and baby	63	84.76	12.68	50.00	80.00	80.00	100.00	100.00
Post: HIV in mother and baby	59	92.88	14.51	0.00	90.00	100.00	100.00	100.00
Difference: HIV in mother and baby	59	7.97	20.58	−100.00	0.00	10.00	20.00	50.00
Pre: Total	63	76.39	8.81	52.50	70.00	77.50	82.50	92.50
Post: Total	59	89.11	7.34	60.00	85.00	90.00	92.50	100.00
Difference: Total	59	12.37	11.25	−30.00	6.25	12.50	20.00	32.50

HIV, human immunodeficiency virus; s.d., standard deviation.

**FIGURE 1:**
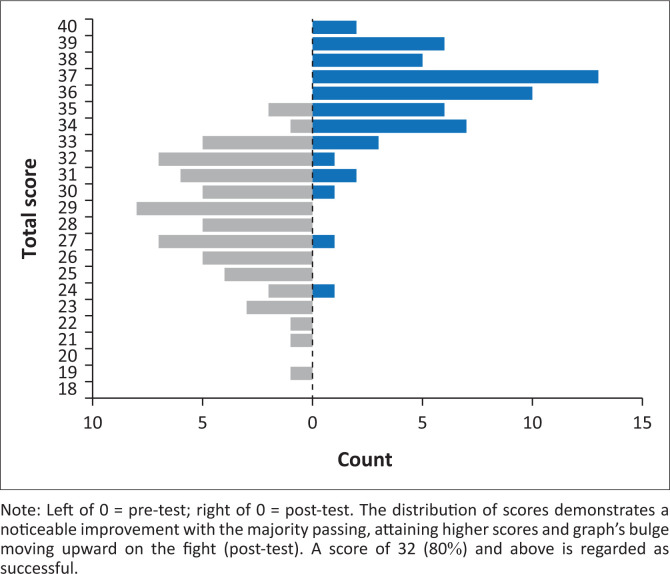
Total average score distribution, pre- and post-tests.

Figure 1 shows the score distribution with the total average pre- and post-test scores, where 40 is the maximum score possible.

**FIGURE 2:**
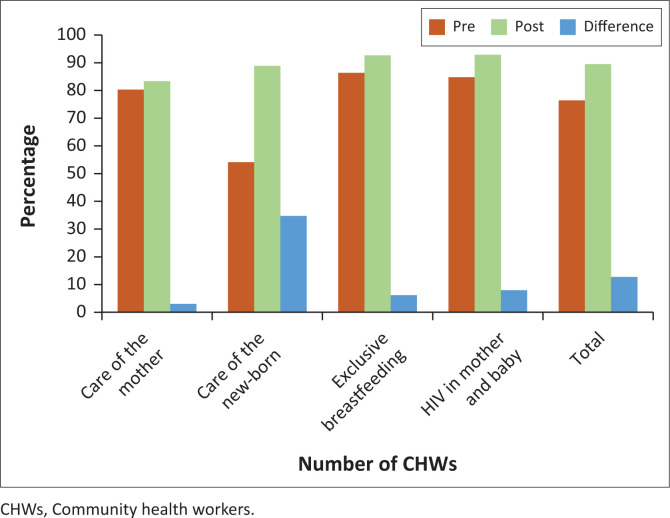
Scores for pre-and post-tests with a recorded difference.

Figure 2 shows the average score for each of the four modules, pre- and post-tests as well as their difference.

Inferential statistics included the test statistics (either *t*-test or Chi² test value), the degrees of freedom (*df*), the *p*-value and the appropriate practical significance (effect size) statistics (Cohen’s *d*) for t-tests and Cramér’s *V* for Chi² tests.

A *p*-value of less than 0.05 indicates significance. Cohen’s *d* is the statistics used to determine the practical significance, if the *p*-value is less than 0.05 for an inferential statistics test based on sample mean values, for example, *t*-test and Scheffé test.^12^ Interpretation intervals are contained in Table 3.

**TABLE 3:** Interpretation intervals for Cohen’s d.

Significance	Interval
Not	*d* < 0.20
Small	0.20 ≤ *d* < 0.50
Medium	0.50 ≤ *d* < 0.80
Large	*d* ≥ 0.80

*Source:* Gravetter FJ, Wallnau LB. Statistics for the behavioral sciences. 8th ed. Belmont, CA: Wadsworth, p. 264 & 628.

Table 4 applies the data, determining *t*-test, *p*, and Cohen’s *d* values.

**TABLE 4:** Matched pair *t*-tests: Pre- versus post-tests.

Module (pre-test, post-test and difference)	Mean	s.d.	*t*	*p*	*df*	*d*
**Care for the mother (*n* = 63)**						
Pre	80.32	13.19	1.36	0.180	-	0.17
Post	83.33	12.95	-	-	62	Not
Diff.	3.02	17.66	-	-	-	-
**Care for the newborn (*n* = 63)**						
Pre	54.13	10.72	19.02	**< 0.0005**	-	**2.40**
Post	87.46	10.62	-	-	62	**Large**
Diff.	33.33	13.91	-	-	-	**-**
**Exclusive breastfeeding (*n* = 60)**						
Pre	86.50	14.59	3.82	**< 0.0005**	-	**0.49**
Post	92.67	8.41	-	**-**	59	**Small**
Diff.	6.17	12.50	-	**-**	-	**-**
**HIV in mother and baby (*n* = 59)**						
Pre	84.92	12.92	2.97	**0.004**	-	**0.39**
Post	92.88	14.51	-	**-**	58	**Small**
Diff.	7.97	20.58	-	**-**	-	**-**
**Total (*n* = 59)**						
Pre	76.74	8.84	8.45	**< 0.0005**	-	**1.10**
Post	89.11	7.34	-	-	58	**Large**
Diff.	12.37	11.25	-	-	-	-

Note: Data set in bold represent matched-pair t-Tests: pre versus post.

*t, t*-test; *p, p*-value; *d*, Cohen’s *d*; Diff., difference; *df*, degree of freedom.

Cramér’s *V*, as shown in Table 5, is the statistics used to determine the practical significance, if the *p*-value is less than 0.05 for an inferential statistics test based on sample frequencies, for example Chi² test.^12^

**TABLE 5:** Interpretation intervals for Cramer’s *V*.

Significance	*df*† = 1	*df*† = 2	*df*† ≥ 3
Not	*V* < 0.10	*V* < 0.07	*V* < 0.06
Small	0.10 ≤ *V* < 0.30	0.07 ≤ *V* < 0.21	0.06 ≤ *V* < 0.17
Moderate	0.30 ≤ *V* < 0.50	0.21 ≤ *V* < 0.35	0.17 ≤ *V* < 0.29
Large	*V* ≥ 0.50	*V* ≥ 0.35	*V* ≥ 0.29

*Source:* Gravetter FJ, Wallnau LB. Statistics for the behavioral sciences. 8th ed. Belmont, CA: Wadsworth, p. 264 & 628.

*df*, degree of freedom.

† , Minimum (number of rows, number of columns) = 1.

The null hypothesis for each of the Chi² tests is that the percentage of participants with a score ≥ 80% for the post-test group is equal to the percentage for the pre-test group. Chi² tests have been used to determine whether the pass rate (pass if score ≥ 80%) improved from pre- to post-test. See Table 6 for the frequency distribution and Chi_2_ results. It is interesting to note that the *t*-test result for Care for the Mother was not significant (*p* = 0.180), whilst the Chi² result for this variable was significant (*p* = 0.012, *V* = 0.32).

**TABLE 6:** Frequency distribution and Chi^2^ tests: Pre and post scores more than 80%.

Module (pre- and post-test)	*n*	80%+		Difference (%)	Chi²	*p* (*df* = 1)	*V*
		Pre-test	Post-test (%)				
**Care of the Mother**							
Pre	63	45	71.4	14.3	6.30	**0.**012	**0.32 Medium**
Post	63	54	85.7	-	-	-	
**Care of the Newborn**							
Pre	63	2	3.2	88.9	1619.41	**< 0.0005**	**5.07 Large**
Post	63	58	92.1	-	-	**-**	
**Exclusive Breastfeeding**							
Pre	63	50	79.4	17.3	10.97	**0.001**	**0.43 Medium**
Post	60	58	96.7	-	-	**-**	
**HIV in Mother and Baby**							
Pre	63	51	81.0	14.0	7.46	**0.006**	**0.36 Medium**
Post	59	56	94.9	-	-	**-**	
**Total**							
Pre	63	26	41.3	50.3	61.48	**< 0.0005**	**1.02 Large**
Post	59	54	91.5	-	-	-	

*Note*: Data set in bold represent matched-pair t-Tests: pre versus post.

*df*, degree of freedom.

The results indicate that most CHWs had good prior knowledge of maternal care, information related to HIV and exclusive breastfeeding, with pre-test scores of 80% or more. The programme manager indicated that training in Growth Monitoring and Nutrition had been convened for CHWs previously by a dietician who had focussed on exclusive breastfeeding. Knowledge of these topics was confirmed in discussion with CHWs.

They had been recruited earlier to a programme sponsored by a funding agency which aimed to reduce transmission of HIV from mothers to babies. Hence, their baseline knowledge as evidenced in the pre-testing on Care of Mothers, Exclusive Breastfeeding and HIV in Mother and Baby was good. The CHWs had less prior knowledge of caring for newborn babies. As a result, the difference between pre-test and post-test scores for these modules was the greatest.

### Qualitative results

Focus group discussions and interviews with CHWs and other key stakeholders provided the data to understand CHW response and attitude to the learning programme, and the factors promoting and inhibiting their learning. The following themes and sub-themes were elicited (Table 7):

**TABLE 7:** Themes and sub-themes.

Themes	Sub themes
The value of learning	Gaining knowledge improved levels of confidence Improved knowledge meant improved feelings of competence Learning improves motivation and inspires to achieve more
The value of the Skills2Care method and process	The Skills2Care method made learning accessible Learning material was compiled in an accessible and simple way Organising in groups facilitated learning WhatsApp groups had not been essential to learning
Factors that enabled learning	Facilitation of the process was beneficial An encouraging, participatory, adult-education approach promoted learning Support of nurses at the clinics assisted learning (and was appreciated) Following the pre-determined method was effective A certificate for course completion added incentive to learning
Factors that inhibit learning	Home circumstances

### The value of learning

Community health workers valued the learning process for their improved knowledge, which in turn gave them more confidence and competence. They responded well to the participatory approach to learning.

#### Gaining knowledge improved levels of confidence

Community health workers considered that they were gaining valuable knowledge which made them feel more confident in their roles. They considered it useful and empowering:

‘It was good to study so that you can know a lot. Then when I see my clients, I feel that I can tell them information that I have learned’. (CHW, female, 26 years old)

‘Sometimes community members will come to your house. They want you to help them. So, these modules give you more confidence to know the right answers for them. It improves your attitude too because you know you are right’. (CHW, female, 42 years old)

Community health workers repeatedly acknowledged that community members did not differentiate between themselves and the professional nurses. They explained that whilst their specific role had been explained to communities, their uniform and association with the clinic meant that their distinction as being CHWs was lost. Being equipped with improved knowledge made them more comfortable to better support communities whose expectations exceeded their competencies and qualifications:

‘They don’t see us as community care workers. They see us as nurses, even if we explain to them. So, they are always asking questions, like “Why are my feet swollen when I am pregnant?” Now we know and can assess and tell them that they must go to the clinic. So, it is important for us to study’. (CHW, female, 34 years old)

#### Improved knowledge meant improved feelings of competence

Community health workers described feeling empowered by the learning: more competent and better equipped to help their clients and support their communities with their improved knowledge:

‘The book has the information. So, we learn about theory. We are very happy to have that book and to read and get knowledge. Although we are practical at the clinic, but we have to have theory … The book really helped.’ (CHW, male, 22 years old)

‘We are comfortable now to go to visit at the homes, because we are more confident that we know our work.’ (CHW, female, 24 years old)

‘Their learning makes them competent. They know they can give the right advice, help their clients and refer back to the clinic or hospital when it is necessary.’ (Manager, male, 40 years old)

‘Building on their [*CHW*] knowledge is empowering. It means they never feel undermined. Building their confidence is also important to the learning. It is always exciting to watch people grow in their knowledge and confidence’. (Facilitator, female, 44 years old)

#### Learning improves motivation and inspires to achieve more

Community health workers described feeling more motivated going to households armed with knowledge:

‘We are now excited to go to households. I wake up and feel motivated. I don’t feel anxious like I would sometimes feel. Especially with the mommies and their babies’. (CHW, female, 33 years old)

Some CHWs indicated that they would like to continue to learn, and Skills2Care provided the motivation and inspiration to do so:

‘When you are learning, you are growing. When you are growing, you are feeling stronger. You want to learn; you want to be the best you can. You can always be better so that motivates you to learn’. (CHW, female, 28 years old)

‘At the end, you can be a nurse. That studying motivates you to consider learning more. It encourages us’. (CHW, female, 26 years old)

‘I see myself one day as a professional nurse. It was so helpful. Nothing seemed too hard. This makes me to believe that I can become a professional nurse one day’. (CHW, female, 26 years old)

### The value of the Skills2Care method and process

#### The Skills2Care method made learning accessible

Community health workers and stakeholders noted that the method of learning with question and answer content, case-studies and self-tests served to promote learning. The discussions that were held were interesting and informative. The case studies were useful and CHWs enjoyed reading about the challenges of other CHWs:

‘I used question and answer method throughout and together we worked through the material. You need to know facilitation. Then you make the CHWs [*community health workers*] to think. Encouraging participation and discussion builds the confidence of CHWs [*community health workers*] to believe in themselves. It helped to motivate them to learn’. (Facilitator, female, 44 years old)

Following the pre-determined learning process was effective. The facilitator indicated that it was important to follow the process as determined by the Skills2Care manual:

‘The manual has a prescribed process. It is systematic. It has been tested with other health professionals. It worked well with the CHWs too’. (Facilitator, female, 44 years old)

#### Learning material was compiled in an accessible and simple way

The accessible language and format of the manual lent itself to learning. All CHWs were isiXhosa first-language speakers. Not all were fluent in English, yet repeatedly they stated that the Mother and Baby Care manual was accessible, with many indicating that they preferred to learn in English. They considered that it was written in a style that did not compromise on necessary and essential information:

‘This book was simplified. It made learning much easier. The clinic nurses helped us too. But the book gave us information that we didn’t know. It encourages us’. (CHW, female, 44 years old)

‘There was nothing that was too hard. It was simple, simple, simple English’. (CHW, female, 29 years old)

‘That book, it was so helpful to know. I enjoyed it. I like to learn and to feel that I am getting better to help my clients’. (CHW, female, 35 years old)

‘The case studies were an example of what the book talked about. They applied the knowledge from the book’. (CHW, female, 42 years old)

The self-assessment test at the end of the modules provided an opportunity for checking their knowledge. The opportunity to test their knowledge was appreciated by the CHWs:

‘I liked the tests at the back of the book. They motivated me to learn. I could tell how much I was gaining’. (CHW, female, 26 years old)

#### Organising in groups facilitated learning

Most CHWs reported enjoying working together with their colleagues in groups as it motivated learning.. They were able to consider ideas and raise challenges amongst peers in a non-threatening environment:

‘Actually, we came to enjoy the groups. We liked to get together. We shared our stories, successes and our failures, so it became a group of friends that we started to trust. So, learning became something to be enjoyed’. (CHW, female, 22 years old)

Beyond the sessions with the facilitator, CHWs often studied together in groups. Groups were held during lunch hours and sometimes when the clinic was deemed to be ‘quiet’. Mostly, group sessions lasted about an hour.

Collectively, CHWs were able to work through and learn the material, and considered this time beneficial:

‘We were four in our clinic. We would come together on Fridays and learn together. If there was spare time at the clinic, we would go to the sister and ask for time to learn. It made us to know the manual well. We would talk to each other and encourage each other to learn’. (CHW, female, 27 years old)

‘Sometimes you are shy about a term in the book, you are not sure. But in the group, you know your care workers, you feel free. You can ask your questions’. (CHW, female, 23 years old)

‘I liked to learn with my colleagues. Then we develop a way of being together, so we didn’t feel embarrassed to ask questions. We knew that our questions were valuable’. (CHW, male, 25 years old)

#### WhatsApp groups not necessary for learning

Skills2Care suggested the formation of WhatsApp groups to support learning. Many CHWs did not own smartphones or they could not afford to buy data and were therefore unable to access WhatsApp. Its absence seems not to have been a deterrent to learning.

### Factors that enabled Community health worker learning

It was the intangible and unmeasurable factors related to the approach to facilitation, facilitator encouragement and support that appeared to promote and motivate the learning of the CHWs most. Some of these have been described above. The more tangible factors such as a dedicated place to study, a quiet space and the availability of a smartphone to connect to a study group were seemingly less important.

#### Facilitation of the process was beneficial

The learning of the CHW is successful with the support of a facilitator. The need for a facilitator, as compared to a self-study method, had previously been determined by SPF in an earlier pilot study. Key stakeholders confirmed this earlier finding:

‘The facilitator made it easy. Mam G [*facilitator*] asked questions, she got us to think. She always believed we could do the work, even if we didn’t think we could’. (CHW, female, 49 years old)

‘We liked to learn. So, when Mam G came, we would enjoy ourselves. We were learning but we didn’t know we were learning. She made it to feel easy’. (CHW, female, 23 years old)

‘It’s important to have a facilitator. You can make them to participate. You ask them questions. You get them to think, and you tell them about your experiences. You are friendly and you make them to feel comfortable. That is the role of the facilitator. Then they learn’. (Facilitator, female, 44 years old)

#### An encouraging, participatory adult-education approach promoted learning

The facilitator was clear that a participatory, adult education approach was important. She explained that the active participation of the CHWs is a successful with adult learning:

‘It’s about building on the knowledge that adults have. As an adult educator you always assume that the people you are teaching have knowledge. So, you don’t feed them information, it must come from them. When they are told the information, they don’t value the information they have, they are afraid of the information they know’. (Facilitator, female, 44 years old)

The support and encouragement from the facilitator were important:

‘Mam G always believed in us. When we came to difficult topics, then we talked amongst ourselves and she guided us to the right answers. It was nice’. (CHW, female, 28 years old)

The facilitator explained that she needed to be flexible in her approach:

‘We know group discussion will always be effective. But then someone doesn’t follow, and then you use one-on-one method. You can’t be like a professional nurse, standing there and lecturing in front of people. They get drowsy with that approach. Some people like to write, some people like to talk, so you accommodate them, and use all the skills you have’. (Facilitator, female, 44 years old)

‘You explain to them, if you don’t know something, that’s normal. Even the president doesn’t know everything. Everybody must be bright in their corner where they are’. (Facilitator, female, 44 years old)

‘I also keep communication going all the time. My number is available to CHWs. They can call me anytime or send a WhatsApp message. I am available. I call them back as soon as I can’. (Facilitator, female, 44 years old)

#### Support of nurses at the clinics assisted learning (and was appreciated)

At all the clinics, CHWs had encountered professional nurses who had been supportive and helpful. If concepts required clarification, CHWs would ask the nurses. They had been willing to help and provided explanations as required, which was much appreciated:

‘At our clinic, we sit next to the children’s consulting room, and then there’s the ANC [*antenatal clinic*] room. So, the nursing sister, she is always there, and she say to us her door is always open. So, we go to her and ask her. She likes to have us there, so she is there to help us’. (CHW, female, 31 years old)

#### A certificate for course completion adds incentive to learning

Skills2Care is in its development phase and has not yet been accredited. Community health workers received a certificate to indicate course completion:

‘We want to get our certificates. Then we can add them to our CV [*curriculum vitae*] and show that we have done the extra courses. We don’t want to do courses without some recognition for our studies. It makes a difference’. (CHW, female, 27 years old)

‘CHWs [*community health workers*] earn a stipend. It is a small amount that helps them to do the work that is necessary. We need to think of ways in which they can remain motivated. Having training and providing certificates recognises the effort and the work they have put in. It provides some additional tangible way of thanking them. The certificate is so important. Without it, CHWs would feel the training was not worthwhile’. (Facilitator, female, 44 years old)

### Factors that inhibited learning

#### Home circumstances

Whilst the group of CHWs had many similar characteristics, their home circumstances were not homogenous. For some CHWs, finding time to study at home was difficult. Many relied solely on their time at the clinic to study. Those who were able to study at home had made special arrangements such as setting up the family to watch television whilst they studied. Others studied at night, after putting their children to bed. Weekends were generally regarded as a difficult time for studying:

‘I study when it is late, and my children have gone to bed. Sometimes, I am tired, but after they are asleep, it is quiet, and I can sit and work’. (CHW, female, 37 years old)

‘I just study, study, study, especially when it is late, and it is quiet at home. Maybe three times a week, I will study like this’. (CHW, female, 28 years old)

‘You know here in the weekends, it is too, too difficult to study. There are always other things that we have to do. It is funerals to attend, sometimes these are far from here, but we must attend them. Sunday, the day is filled with our church’. (CHW, female, 42 years old)

## Discussion

The study sets out to determine the acceptability and appropriateness of the Skills2Care learning model for CHWs, utilising a decentralised, cooperative learning approach on-site at the health facilities. It was conducted in resource-limited, rural settings. A number of systematic reviews have recently indicated the benefit of ongoing training for CHWs, in improving their knowledge and keeping them relevant and abreast with the recent developments in healthcare. Studies have seemingly not refined their focus on the method of training for CHWs in resource-poor settings. This study has demonstrated a model that is conducted on-site, at the clinics. It requires the support of a facilitator, peer-collaboration and the Skills2Care manual. Its physical requirements are relatively limited and potentially accessible at scale.

Scott et al. indicated that training for CHWs was required to be context specific.^1^ Skills2Care fulfils this criterion as it is aimed specifically at rural and resource-limited settings and offers a programme that can support improvements in the knowledge of CHWs.

Results for the different pre-tests varied substantially, from a high average of 86% for the module on *Exclusive Breastfeeding*, to 54% for the *Care of the Newborn Baby* module. It was clear that the CHWs had prior knowledge in specific areas, especially related to breastfeeding, HIV and care of mothers. Despite their prior learning, there was nonetheless evidence of knowledge improvement in all modules, ranging from 3% to 35%.

Most improvement was evident in the module *Care of the Newborn Baby*. The CHWs had limited prior knowledge of the topic, and their group average score increased from 54% to 89%. These results demonstrate the possibility of introducing new topics for CHW learning and their potential for success. These results were similar to those documented for professional nurses.^13^

Schneider et al. noted that many CHW programmes focussed on maternal and child health.^2^ This programme followed that trend. They emphasised that programmes, having been donor-led, needed to be integrated into the national health programmes and local PHC systems. Whilst this programme has indeed been included in the PHC system, advocating for its implementation at provincial and national departments with emphasis on local needs is essential if it is to be implemented more widely.

Whilst prior Skills2Care experience had shown that the facilitator was integral to the process,^14^ this study confirmed the nature of the facilitation being important. An understanding of the principles of adult education, adopting an encouraging approach with CHWs and nurturing participation were deemed important. The attitude of the professional nurses in this study indicated their willingness to support CHW learning. They may well provide a clue to the necessary support for CHW learning at health facilities in the future.

Gaining new knowledge provided CHWs with a sense of confidence and improved their competence. This newly gained competence is ‘an important driver to empowerment’.^15^ Community health workers hope and belief in themselves and their ability, triggered their thinking towards further learning and their career development.

That CHWs reported valuing the programme and enjoyed the learning was encouraging and suggests that this method of continuing education may be as successful with CHWs as it has been with professional nurses and midwives.

The recent Oxfam report on health workers^16^ and the chapter by Vale and Di Maurio^17^ both attest to the value that CHWs bring to the health system. Both argue that their intimate knowledge of local communities and of the households within the communities was invaluable yet undervalued by the health system. Empowering CHWs with knowledge and understanding may contribute to building their confidence and providing an avenue in which CHWs are emboldened to speak up and challenge the system so as to better understand and appreciate the value that they offer the health system.

The evidence gathered in this study points to the programme’s appropriateness for improved CHW knowledge, and the results demonstrate that the model has been successful. It suggests the value of using a decentralised, group learning approach on a larger scale. The Skills2Care method can possibly be applied in all settings, not just rural.

The small study sample with only four modules for learning in this study were its main limitations. It nevertheless provides some insight into what may be possible with a larger programme. It holds the promise of expanded programmes of learning for CHWs with larger numbers and a wider range of topics. The study did not examine the costs and cost-effectiveness of the programme in a rural setting.

## Conclusion

Community health workers are integral to health systems, and PHC especially in low- and middle-income countries. Their ongoing training needs to be appropriate, affordable and prioritised if they are to be properly recognised by the health system and their benefit are to be maximised. This study showed that the on-site, cooperative Skills2Care learning programme, with the support of a facilitator, was a success and well received by the CHWs. A comparison of pre- to post-test results demonstrated a significant improvement in cognitive knowledge with three of the four modules. It showed that a participatory approach to facilitation to support and encourage CHW learning is important. Health facilities in remote, rural areas in particular may benefit from the Skills2Care programme. The engagement of nursing staff at the clinics may provide further support for continuing education for CHWs. Advocacy within the Health Department for the ongoing implementation of this project will enable it to realise its potential.

## References

[1] Scott K, Beckham S, Gross M, et al. What do we know about community-based health worker programs? A systematic review of existing reviews on community health workers. Hum Resour Health. 2018;16:39. 10.1186/s12960-018-0304-x 30115074 PMC6097220

[2] Schneider H, Okello D, Lehmann U. The global pendulum swing towards community health workers in low- and middle-income countries: A scoping review of trends, geographical distribution and programmatic orientations, 2005 to 2014. Hum Resour Health. 2016;14(1):65. 10.1186/s12960-016-0163-2 27784298 PMC5081930

[3] Kok MC, Kane SS, Tulloch O, et al. How does context influence performance of community health workers in low- and middle-income countries? Evidence from the literature. Health Res Policy Syst. 2015;13:13. 10.1186/s12961-015-0001-3 25890229 PMC4358881

[4] World Health Organisation. Community health workers delivering primary health care: Opportunities and challenges. A resolution of the seventy -second world health assembly. Agenda item 11.5. Geneva: WHO; 2019.

[5] Lehmann U, Sanders D. Community health workers, what do we know about them? The state of the evidence of programmes, activities, costs and impact on health outcomes using community health workers. Bellville, Cape Town: School of Public Health, University of Western Cape.

[6] Murphy J, Moolla A, Kgowedi S, et al. Community health worker models in South Africa: A qualitative study on policy implementation of the 2018/19 revised framework. Health Policy Plan. 2021;36(4):384–396. 10.1093/heapol/czaa172 33367608 PMC8128020

[7] Glenton C, Colvin CJ, Carlsen B, et al. Barriers and facilitators to the implementation of lay health worker programmes to improve access to maternal and child health: Qualitative evidence synthesis. Cochrane Database Sys Rev. 2013;10:CD010414. 10.1002/14651858.CD010414.pub2 PMC6396344 24101553

[8] Woods D. Mother and baby care for community health workers. Bettercare. No date. Available from: https://bettercare.co.za/learning-programmes/mother-and-baby-care-for-community-health-workers/

[9] Woods DL & Theron GB. The impact of the Perinatal Education Programme on cognitive knowledge in midwives. S Afr Med J. 1995;85:150–153. 7777961

[10] South African National Health Department of Health. Policy framework and strategy for ward based primary health care outreach teams. Pretoria: NDoH; 2018. Available from: http://www.health.gov.za/wp-content/uploads/2020/11/policy-wbphcot-4-april-2018_final-copy.pdf

[11] Pillay Y, Barron P. The implementation of PHC re-engineering in South Africa: PHASA newsletter 15 November 2011 [homepage on the Internet]. Available from: http://www.phasa.org.za/articles/the-implementation-of-phc-re-engineering-insouth-africa.html

[12] Gravetter FJ, Wallnau LB. Statistics for the behavioral sciences. 8th ed. Belmont, CA: Wadsworth, p. 264 & 628.

[13] Woods DL, Greenfield DH. Teaching in under-resourced hospitals: Experience from South Africa. NeoReviews. 2010;11:5–11. 10.1542/neo.11-1-e5

[14] Boulle T. Report on Skills2Care Programme of learning. Nelson Mandela Bay. SPF internal report.

[15] Kane S, Kok M, Ormel H, et al. Limits and opportunities to community health worker empowerment: A multi-country comparative study. Soc Sc Med. 2016;164:27–34. 10.1016/j.socscimed.2016.07.019 27459022

[16] Oxfam, South Africa. The right to dignified healthcare work is a right to dignified healthcare for all. No date [cited 2021 Sept 30]. Available from: https://www.oxfam.org.za/wp-content/uploads/2020/07/Oxfam_Care4Carers-Report_Final_20200701.pdf

[17] Vale B, Di Paola M. Knowledge, power and the role of frontline health workers for South Africa’s epidemic preparedness. In: Zamanzima M, editor. Epidemics and the health of African nations, Chapter 8. South Africa: Jacana Media, 2019; p. 229–269.

